# Challenges in Early Years data and their implications on programme evaluations

**DOI:** 10.23889/ijpds.v9i5.2773

**Published:** 2024-09-18

**Authors:** Silvia Colonna, Kathryn Helliwell

**Affiliations:** 1 Welsh Government

The Welsh Government funds a wide range of Early Years programmes to both support parents to remain in work and children’s social, emotional and educational development. The interest of Welsh Ministers in this policy area has grown in recent years with expansions in this area carefully considered. The potential for expanding the offer has generated a greater need for evidence to understand the effectiveness of the delivery against the proposed objectives.

Data linking is a valuable and efficient way to provide complex evaluations of policy programmes as it maximises the use of existing data sources without increasing the burdens on data providers and provides comprehensive information that would otherwise be limited with a single dataset.

However, Welsh Government research on programme evaluation in Early Years is facing challenges and this work aims at providing an overview of these issues, their impact on Early Years analytical work and progress made to address them. In particular, the main challenges are related to data quality and data availability that stem from lack of metadata and contextual information, poor technological infrastructure and, at times, a disconnect between policy / operational delivery and data collection. Progress is being made to address these challenges, such as acquiring new datasets or improving the quality of existing ones but these developments take time. At the same time as helping to tackle these structural challenges, the Early Years analytical team is exploring a range of novel approaches to provide robust evaluation of Early Years interventions despite the data issues.

